# Orchestration of the tumor microenvironment by citrus flavonoids: from preclinical mechanisms to translational therapeutic

**DOI:** 10.3389/fimmu.2026.1806088

**Published:** 2026-05-04

**Authors:** Siyao Ma, Xuebing Wang, Mingzhu Li, Wenping Wang, Yanyi Ren, Yue Shen, Ke Yang, Ze Zhang, Zitong Feng, Shuhan Tang

**Affiliations:** 1 The First Clinical College, Liaoning University of Traditional Chinese Medicine, Shenyang, China; 2 Liaoning Cancer Hospital and Institute, Cancer Hospital of Dalian University of Technology, Shenyang, China

**Keywords:** Citri Reticulatae Pericarpium (CRP), clinical translation, flavonoids, lung cancer, narrative review, tumor microenvironment

## Abstract

Malignant lung tumors are the leading cause of cancer-related mortality worldwide, and therefore remodeling of the tumor microenvironment (TME) has become an important strategy to overcome anti-tumor therapy resistance in lung cancer. Flavonoid components isolated from Citri Reticulatae Pericarpium (CRP), such as nobiletin, hesperidin, and tangeretin, have been shown to modulate the lung cancer TME in a highly relevant manner. The present narrative review collected literature from the PubMed, Web of Science, Embase, CNKI, and Wanfang databases between 2016 and 2026, and hence discussed the molecular mechanisms by which CRP flavonoids reshape the lung cancer TME, namely their regulation of oxidative stress-inflammation homeostasis, correction of lipid metabolic reprogramming, induction of pyroptosis, and inhibition of epithelial-mesenchymal transition. Discussion of the role of EMT and tumor angiogenesis suppression were also presented. Then evidence regarding the modulation of emerging targets was introduced, namely ferroptosis and the cyclic GMP-AMP synthase-stimulator of interferon genes (cGAS-STING) pathway, which are both promising targets in lung cancer models. Translational prospects of CRP flavonoids were led to enhancing immune checkpoint inhibitor (ICI) efficacy and developing nano-delivery systems. The article first outlined the fundamental barriers, then gave a very systematic and critical review of the contradictory findings, context-dependent effects, and methodological limitations in the existing literature; and then pointed out the gaps in frontier research. Therefore, it provides an excellent theoretical foundation for the research and development of anti-lung cancer drugs from CRP flavonoids, while also objectively identifying the current knowledge gaps and clinical translation bottlenecks.

## 1 Introduction

Lung cancer is the leading cause of cancer mortality worldwide, accounting for 2.2 million new cases and 1.8 million deaths in 2022 (1, 2). The tumor microenvironment (TME) has been clearly and convincingly established as a critical driver of lung cancer progression and anti-cancer treatment resistance, with major factors including immunosuppressive cell infiltration, chronic inflammation, aberrant angiogenesis, and extracellular matrix remodeling (3, 4). Therefore, therapy targeting the TME to reverse immune evasion and improve treatment efficacy is a very active and promising direction in precision medicine for lung cancer.

Citri Reticulatae Pericarpium (CRP), the dried, mature outer peel of Citrus reticulata Blanco and its cultivated varieties from the Rutaceae family, was first recorded in the Shen Nong Ben Cao Jing (Shennong’s Classic of Materia Medica). The well-established traditional indications involves regulating qi, strengthening the spleen, drying dampness, and resolving phlegm (5). As a result, it is one of the most commonly used herbs in clinical practice, and has been employed for respiratory disorders and has well-documented applications in compound formulas for pulmonary malignancies (6–8). CRP is derived from natural product sources, and its bioactive constituents have favorable toxicity profiles and potent immunomodulatory properties, hence it has attracted growing interest in lung cancer TME research. The flavonoid fraction is the primary material basis for CRP’s antineoplastic activity, containing nobiletin, hesperidin, tangeretin, naringin, sinensetin, and other flavonoids, with polymethoxyflavones (PMFs) being its most characteristic components (9).

Since systematic theoretical frameworks for CRP flavonoid modulation of lung cancer TME are still far from complete. It is appropriate to point out first that most previous studies have examined single signaling axes, hence the integrative mechanisms of pathway crosstalk remain undefined, and therefore the “multi-component, multi-target, multi-pathway” hypothesis lacks of experimental support. The second point to be made is that the investigation of emerging TME regulatory nodes, namely ferroptosis and the cGAS-STING pathway, represents an important gap in current CRP flavonoid anti-lung cancer research. The third point is that the core components have poor aqueous solubility, significant first-pass metabolism, and therefore limited systemic exposure.

To bridge these research gaps, the narrative review systematically retrieved literature from Public MEDLINE (PubMed), Web of Science, Excerpta Medica Database (Embase), China National Knowledge Infrastructure (CNKI), and Wanfang databases covering the period 2016–2026 to outline the molecular mechanisms by which CRP flavonoids intervene in lung cancer by regulating the TME. And then followed by an analysis of translational potential and central barriers of the clinical translation, which aim to build a theoretical basis for collaborative modulation of lung cancer TME of CRP flavonoid, and to provide clear direction for antineoplastic drug development from natural product origins and identifies priority areas for future research.

## 2 Molecular characteristics and anticancer activity of CRP flavonoids

CRP abounds in flavonoid constituents, which tumor-suppressive efficacy varies with geographic origin and storage duration. With chemical analysis and bioactivity assays, the structural features of CRP flavonoids and their roles in cancer growth intervention have been gradually illustrated. This section summarizes research progress on chemical character analysis, evaluation of anti-inflammatory and anticancer activities, and intestinal absorption mechanisms.

Some researchers (10) collected twenty-one CRP samples of various ages and geographic origins, from which ethyl acetate extracts were prepared by solvent extraction, high-performance liquid chromatography (HPLC) was used for flavonoid chemical profiling, and the half maximal inhibitory concentration (IC_50_) was employed to evaluate the bioactivity of flavonoid compounds. After which Hesperidin, nobiletin and tangeretin and 3,5,6,7,8,3’,4’-heptamethoxyflavone were detected in all specimens, and the Sichuan Dahongpao sample showed the best anti-inflammatory activity (IC_50_=10.37 μg·mL^-^¹), while the Guangdong Xinhui specimen had the strongest antiproliferative effect against A549 lung carcinoma cells (IC_50_=39.55 μg·mL^-^¹). Most importantly, correlation analysis clearly established a strong positive relationship between nobiletin content and anti-inflammatory potency, and the sample with the highest nobiletin content also suppressed A549 cell migration most effectively and induced apoptosis most robustly. Therefore, nobiletin is an excellent candidate for quality control of CRP materia medica. Details are shown in **Table 1**.

**Table 1:** Molecular characteristics and anticancer activity of CRP flavonoids.

Compound	Molecular formula	Molecular weight (g·mol^-^¹)	Structural features	Content in CRP	Plant part	Isolation method	Clinical application	References
Nobiletin	C_21_H_22_O_8_	402.4	5,6,7,8,3’,4’-Hexamethoxyflavone	0.02%-0.65%	Pericarp	95% ethanol reflux (1:10, 20min×3) → petroleum ether/ethyl acetate partition	Extensive	(10–12)
Sinensetin	C_20_H_20_O_7_	372.37	5,6,7,3’,4’-Pentamethoxyflavone	Not specified			Moderate	(10–12)
Naringin	C_27_H_32_O_14_	580.54	Naringenin-7-O-neohesperidoside	2.11%-9.11%			Moderate	(10–12)
Hesperidin	C_28_H_34_O_15_	610.56	Hesperetin-7-O-rutinoside	2.11%-9.11%			Extensive	(10–12)
Hesperetin	C_16_H_1>4_O_6_	302.28	5,7,3’-Trihydroxy-4’-methoxyflavanone	Not specified			Limited	(10–12)
Tangeretin	C_21_H_22_O_8_	372.37	5,6,7,8,4’-Pentamethoxyflavone	0.01%-0.58%			Limited	(10–12)
Naringenin	C_15_H_12_O_5_	272	4’,5,7-trihydroxyflavanone	Not specified			Limited	(10–12)

Thirteen batches (11) of CRP samples from different origins and with different storage durations were collected and tested for IC_50_ determination against A549 lung cancer cells, yielding inhibitory concentrations in the range of 10.53 to 53.75 mg·mL^-1^. Research has shown that the 2018 Hubei specimen had the strongest suppressive activity (IC_50_=10.53 ± 0.48 mg·mL^-1^), whereas the 2020 Hubei sample was the least active (IC_50_=53.75 ± 3.86 mg·mL^-1)^, as shown in **Table 1**. From the intra-regional temporal comparison, it was clearly and convincingly shown that the 2010 sample from Tianma Village, Xinhui District, Guangdong Province (IC_50_=18.18 ± 1.71 mg·mL^-1)^ was superior to its 2015 counterpart (IC_50_=21.25 ± 1.83 mg·mL^-1^). Likewise, the 2018 Hunan specimen (IC_50_=16.53 ± 1.87 mg·mL^-1)^ had much greater activity than the 2020 Hubei sample. The authors convincingly implicate storage duration as a determinant of antineoplastic potency and provide clear quantitative data for three major flavonoid constituents, followed as hesperidin 2.11%-9.11%, nobiletin 0.02%-0.65%, tangeretin 0.01%-0.58%. Both provenance and vintage were shown to affect chemical composition. The 2020 Kaiping, Guangdong sample had maximal hesperidin content (6.93%) without a corresponding increase in nobiletin (0.48%) or tangeretin (0.45%). In contrast, the 2018 Hunan specimen had elevated nobiletin (0.65%) and tangeretin (0.58%) along with the lowest IC_50_ (16.53 mg·mL^-1)^, thereby directly supporting the hypothesis that higher nobiletin and tangeretin concentrations are associated with greater antineoplastic activity.

Additional investigators (12) established C57BL/6 murine subcutaneous lung cancer xenograft models and Caco-2/HepG2 co-culture systems to study the fluctuations in CRP flavonoid antineoplastic activity against non-small cell lung cancer and their molecular mechanisms, with untargeted metabolomic profiling, which revealed fifteen signature differential metabolites as the direct material basis for activity variation during extract storage. From the analytical data, it was clearly established that nobiletin and tangeretin concentrations determine the post-absorptive antineoplastic potency of samples from different storage vintages. And subsequent mechanistic studies showed that extracts of all storage durations exerted antineoplastic effects by inducing reactive oxygen species (ROS) accumulation-mediated apoptosis and G2/M phase cell cycle arrest, both of which involve activation of the mitogen-activated protein kinase (MAPK) pathway. Experimental studies on intestinal absorption mechanisms have clearly demonstrated that nobiletin and tangeretin suppress P-glycoprotein efflux function. Since P-glycoprotein is an adenosine triphosphate-binding cassette transporter located on the apical surface of intestinal epithelial cells and functions to pump substrates back into the intestinal lumen, inhibition of this transporter therefore promotes greater absorption. Regarding intestinal absorption mechanisms, the entry of therapeutic agents into systemic circulation can be explained. Nobiletin and tangeretin mediate P-glycoprotein suppression, thereby facilitating increased translocation of hesperidin, isohesperidin, nobiletin, and tangeretin into the blood circulation. Since translocation quantity directly reflects intestinal absorption efficiency, it is reasonable to define intestinal absorption as the amount of drug that actually crosses the intestinal barrier, enters epithelial cells and reaches the systemic circulation. Multivariate analysis clearly identified constituent translocation as the most important discriminator among CRP flavonoid extracts of different storage vintages. Moreover, the translocation quantities of hesperidin, nobiletin, and tangeretin were strongly and consistently correlated with post-absorptive suppressive activity, with higher translocation predicting greater antineoplastic efficacy.

These three studies are closely interrelated, while focus on different aspects. Study (10) first employed HPLC for flavonoid chemical profiling, and compared the anti-inflammatory and antineoplastic activity differences of CRP flavonoids from various origins, and proposed nobiletin as a key marker for quality control. Study (11) analyzed CRP samples of different batches, geographic origins, and storage durations, revealing a positive correlation between storage time and antineoplastic potency, and established that higher concentrations of flavonoids correspond to stronger antineoplastic activity. Study (12) utilized animal models and metabolomics techniques to demonstrate that greater translocation predicting better anti-lung cancer efficacy. However, there are many limitations in existing researches. First, sample sources are concentrated in southern Chinese production regions, which constrains the findings of other areas. Second, *in vitro* activity are only rely on the A549 cell line, without comparing with other lung cancer cell controls. Third, animal experiments lack positive control groups, making it difficult to assess the relative pharmacological potency. Fourth, storage time is a variable influenced by temperature, humidity, illumination, and other factors, yet current studies have not conducted single-factor or multi-factor analyses. Therefore, subsequent research should expand sample sources to cover more production regions; and employ multiple lung cancer cell controls; introduce positive drug controls in animal experiments; and designing experiments with strictly controlled temperature, humidity, and other variables to clarify optimal storage conditions.

## 3 Mechanism of action of CRP flavonoids

The traditional extraction techniques for CRP flavonoids primarily rely on organic solvents such as methanol and ethanol, often assisted by heat reflux extraction or Soxhlet extraction (10). The major compounds obtained include hesperidin, neohesperidin, nobiletin, and tangeretin, which play a role of anti-lung cancer pharmacological by modulating inflammation, oxidative stress, lipid metabolism, pyroptosis, and epithelial-mesenchymal transition (EMT). This section will further systematically elaborate on their molecular regulatory mechanisms, providing a scientific basis for modern pharmacological research and quality control of CRP.

### 3.1 Oxidative stress and inflammatory modulation

Evidence from a number of studies showing that the flavonoid constituents of CRP have potent suppressive effects on pro-inflammatory mediators, hence their role in inflammatory regulation and cancer prevention is well supported experimentally (13).

Hesperidin (13), a flavonoid from CRP, has well-documented anti-lung cancer effects that are mediated by its antioxidant activity and ability to inhibit tumor cell proliferation, and these effects have been confirmed in animal studies. Specifically, in the Benzo(a)pyrene-induced Swiss albino mice lung cancer model, administration of hesperidin at 25 mg·kg^-^¹ body weight increased the activities of superoxide dismutase, catalase, and glutathione peroxidase, along with the levels of reduced glutathione, vitamin E, and vitamin C, while simultaneously reducing lipid peroxides, carcinoembryonic antigen, several oxidative stress related enzymes, serum aryl hydrocarbon hydroxylase, γ-glutamyl transpeptidase, 5’-nucleotidase, and lactate dehydrogenase. Histopathological analysis and proliferating cell nuclear antigen immunostaining both directly supported its antiproliferative action.

Tangeretin (14), a polymethoxylated flavonoid from CRP, has well-documented antioxidant, anti-inflammatory, and antitumor activities, and its anti-lung cancer effects have been convincingly demonstrated both *in vitro* and *in vivo*. Importantly, polyurethane-induced BALB/c mice treated with tangeretin showed normalized oxidative stress markers, decreased myeloperoxidase activity in lung tissue, and reduced expression of intercellular adhesion molecule-1, the compound interleukin-6, nuclear factor-κB (NF-κB). And By modulating the NF-κB/Intercellular Adhesion Molecule-1 (ICAM-1) and Janus Kinase (JAK)/Signal Transducer and Activator of Transcription 3 (STAT3) signaling pathways, this component thereby decreased the phosphorylation levels of JAK and STAT3, inhibit cancer metastasis, increased caspase-3 expression to promote apoptosis. And its antitumor effect is confirmed by histopathological analysis. *In vitro*, tangeretin suppresses cyclooxygenase-2 (COX-2) induction. The regulation of interleukin-1β (IL-1β)-induced COX-2 expression in A549 cells and constitutive COX-2 expression in H1299 cells was shown by RT-PCR to occur at the transcriptional level. Mechanistic analysis revealed that IL-1β activates Extracellular signal-Regulated Kinase (ERK), p38 MAPK, c-Jun N-terminal Kinase (JNK), and Protein kinase B/AKT (AKT) in A549 cells, and that p38 MAPK, JNK, and PI3K all directly participate in cyclooxygenase -2 induction. Importantly, tangeretin inhibited phosphorylation of p38 MAPK, JNK, and AKT as well as downstream NF-κB activation, thus exerting its anti-inflammatory effect and suppressing lung cancer (15).

Sinensetin (16), a citrus polymethoxyflavone, exhibited very clear and reproducible antitumor activity against non-small cell lung cancer A549 and H1299 cells, as shown by the cell counting kit-8 (CCK8) assay, which gave half-maximal inhibitory concentrations of 81.46 µmol·L^-1^ and 93.15 µmol·L^-1^ respectively. Transwell experiments confirmed that it markedly inhibited cell invasion, while Western blotting and enzyme-linked immunosorbent assay both demonstrated downregulation of N-cadherin and vascular. Sinensetin can also inhibit VEGFA expression, upregulated E-cadherin, enhanced CD8^+^T cell cytotoxicity, and increased the concentrations of Interferon-γ (IFN-γ), IL-2, and Tumor Necrosis Factor-α (TNF-α), and the underlying mechanism was clearly established. Sinensetin suppressed AKT/β-catenin pathway activity, an effect that was reversed by AKT activator SC79 treatment, which in turn reversed sinensetin-mediated suppression of non-small cell lung cancer cell proliferation, invasion, EMT, and immune evasion. *In vivo* experiments then confirmed that sinensetin reduced tumor volume and weight in tumor-bearing mice, downregulated N-cadherin, Vascular Endothelial Growth Factor A (VEGFA), and AKT/β-catenin pathway expression, while upregulating E-cadherin and IFN-γ levels. Therefore, the conclusion is firm and elegant that sinensetin suppresses non-small cell lung cancer cell growth, invasion, EMT, and immune evasion via the AKT/β-catenin pathway.

Naringin (17) and its dextran nanocomposite increased nuclear factor erythroid 2-related factor 2 (Nrf2) expression in diethylnitrosamine/2-acetylaminofluorene induced rat lung cancer models, thereby upregulating glutathione peroxidase and superoxide dismutase activities, raising reduced glutathione content, and lowering lipid peroxidation levels. More importantly, TNF-α, IL-1β, IL-6, NF-κB, and inducible nitric oxide synthase expression were all downregulated, while interleukin-10 levels were elevated, which strongly suggests that naringin exerts lung tissue protective and anticancer effects by modulating the balance of inflammatory factors.

Naringenin (18) exerted clear pro-oxidative stress effects in the A549 cell experiments, as demonstrated by the fact that within the 30-120 µmol·L^-^¹ concentration range, cell proliferation was inhibited, apoptosis was increased, reactive oxygen species levels were elevated, superoxide dismutase activity was reduced, and malondialdehyde content was raised. The text also mentions Nrf2 and its downstream NAD(P)H:quinone oxidoreductase 1 (NQO1). Since heme oxygenase-1 (HO-1) expression was decreased while both P38 MAPK phosphorylation and caspase-3 activation were increased, it was convincingly demonstrated that the constituent induces lung cancer cell apoptosis via the reactive oxygen species/P38-MAPK pathway.

To be noticed, the pro-oxidative stress effect of naringenin is in contrast to the antioxidant, anti-inflammatory, and cytoprotective action of most CRP flavonoids. This effect has been shown to be highly context-dependent, with concentration, compound structure, cellular malignancy, and intracellular redox baseline all serving as potential variables, while none of these hypotheses have been validated by existing studies. Because broad-range concentration gradients were used, it is impossible to determine whether low concentrations have pro-oxidative effects or actually shift toward antioxidant activity. Also, normal bronchial epithelial cell controls are missing, so selective toxicity toward lung cancer cells cannot be conclusively established. Finally, standardized molar concentration comparisons of different flavonoid constituents have not been made. Because the distinction between structure-dependent and dose-dependent effects has not been clearly established, and it remains undetermined whether Nrf2 downregulation is a direct effect of naringenin or a secondary feedback response to oxidative stress damage, the current literature cannot support a systematic analysis of the core mechanisms underlying bidirectional regulation by CRP flavonoid constituents in the lung cancer microenvironment. Therefore, future studies should employ standardized concentration gradients, multiple cell line comparisons, head-to-head monomer constituent comparisons, and rescue experiments to identify effect transition nodes and molecular targets, thereby providing robust evidence for mechanistic elucidation and translational application of CRP flavonoids as lung cancer intervention candidates.

### 3.2 Lipid metabolic reprogramming regulation

Reprogramming of lipid metabolism is a fundamental metabolic feature of tumor cells, serving both to supply energy and biosynthetic precursors for rapid proliferation and to relieve tumor microenvironment stress (19, 20). Disordered lipid metabolism directly connects metabolic dysregulation with immune dysfunction (21), aberrant accumulation of lipid metabolic intermediates impairs immune cell function. Excessive lipid deposition causes reactive oxygen species accumulation, activates inflammasomes, and thereby promotes the release of TNF-α, IL-6, and various other pro-inflammatory mediators, which in turn collaborate to suppress effector T cell infiltration and activity, thus creating an immunosuppressive microenvironment favorable for tumor growth and metastasis (22). Therefore, targeting lipid metabolic reprogramming has become a promising strategy to reverse immune suppression and boost antitumor immunity.

Because CRP-derived flavonoid constituents modulate important lipid metabolic enzymes and transcription factors, they correct the imbalance between lipid synthesis and catabolism, reduce the accumulation of lipotoxic products, and therefore ameliorate the immune microenvironment (23), but the precise mechanisms differ among constituents.

Naringin enhances fatty acid β-oxidation via the AMP-activated protein kinase pathway, suppresses peroxisome proliferator-activated receptor γ transcriptional activity, inhibits lipid synthesis, and thus maintains cholesterol homeostasis (24), while at the same time inhibiting transcription of lipotoxicity-related genes and directly downregulating 3-hydroxy-3-methylglutaryl-coenzyme A reductase (HMGCR), the rate limiting enzyme in cholesterol synthesis (25, 26). Consequently, lipid peroxidation and reactive oxygen species accumulation are reduced, effector T cell function is enhanced, macrophage and neutrophil infiltration is ameliorated (27), metabolic cellular injury is prevented, and finally lung cancer cell proliferation and inflammatory microenvironment formation are both suppressed.

Nobiletin inhibits sterol regulatory element-binding protein-1c (SREBP-1c) mediated fatty acid synthesis hyperactivation by several defined mechanisms. It prevents nuclear translocation of the transcription factor, lowers the protein level and phosphorylation-activated state of its downstream target enzyme acetyl-coenzyme A carboxylase (ACC), thereby blocking fatty acid biosynthesis, and at the same time activates the AMP-activated protein kinase (AMPK) pathway. Because it upregulates carnitine palmitoyltransferase 1 (CPT1) expression and thus promotes fatty acid β-oxidation (28), this molecule reduces intracellular triglyceride and free fatty acid accumulation in tumor cells, decreases lipid availability in the tumor microenvironment, reverses saturated fatty acid induced M2 macrophage polarization, relieves the suppression of CD8^+^T cell proliferation and cytotoxic function (29), and therefore effectively suppresses lung cancer progression and metastasis.

Hesperetin differs from other agents in that it directly and effectively targets cytochrome P450 enzyme-mediated lipid peroxidation and DNA damage: it inhibits Cytochrome P450 1A1 (CYP1A1)/Cytochrome P450 1B1 (CYP1B1) activity, prevents the generation of DNA-damaging reactive intermediates from polyunsaturated fatty acid metabolism, thus lowering gene mutation and tumor initiation risks, while also suppressing interleukin-8 and other inflammatory mediators (30), thereby improving the local immunosuppressive microenvironment and breaking the inflammation - mutation vicious cycle.

### 3.3 Regulation of pyroptosis to remodel TME

Pyroptosis is an inflammatory form of programmed cell death characterized by cell swelling, increased membrane permeability, and release of pro-inflammatory mediators, and it has now been clearly established as a major mechanism of tumor microenvironment remodeling. Specifically, tumor cell pyroptosis induces upregulation of gasdermin B (GSDMB), which in turn activates T cell-mediated antitumor immune responses, thereby creating a positive feedback loop that actively and favorably reshapes the tumor immune microenvironment (31, 32). Among CRP flavonoids, only nobiletin promotes pyroptosis.

Current literature clearly and convincingly demonstrates that nobiletin-induced tumor cell pyroptosis is mediated by the miR-200b/JAZF1/NF-κB pathway. Flow cytometry analysis showed that nobiletin suppresses NF-κB signaling by downregulating miR-200b and upregulating the transcriptional corepressor JAZF1, thereby preventing NOD-like receptor family pyrin domain containing 3 (NLRP3) inflammasome activation and inhibiting the release of interleukin-1β and other pro-inflammatory mediators (33, 34). Nobiletin also promotes Caspase-1/GSDMD-mediated specific pyroptosis, increases CD4^+^ and CD8^+^ T cell infiltration, disrupts the immunosuppressive tumor microenvironment, and ultimately suppresses tumor growth. Researches have shown that the pyroptosis induced by nobiletin is dose-dependent, with a corresponding dose-dependent suppression of tumor cell proliferation (35).

It is appropriate to note that the NLRP3 inflammasome pathway has complex, tumor-immunity-related roles whose regulatory outcomes depend on cell type, tumor type, and host immune status, hence many relevant mechanisms remain to be elucidated. NLRP3 activation in tumor cells can induce pyroptosis, thereby releasing tumor-associated antigens and damage-associated molecular to recruit and activate immune cells. Triggering CD8^+^T cell-mediated antitumor immunity can be effectively achieved by even limited pyroptosis of tumor cells. Some researches results have proved that pyroptosis facilitates the release of pro-inflammatory cytokines and damage-associated molecular patterns (DAMPs), thereby strengthening antitumor immune responses and remodeling the tumor microenvironment (TME). Nevertheless, persistent or abnormally activated pyroptosis may trigger chronic inflammation, radiation-related tissue damage, and even tumor development, thus acting as a double-edged sword, indicating that its clinical application should be approached with caution (36–39).

### 3.4 Regulate epithelial-mesenchymal transition to Remodel TME

EMT dissolves intercellular adhesion by converting epithelial cells into mesenchymal phenotypes, it thereby creates the conditions for tumor invasion and distant dissemination. However, it also engages in crosstalk within the tumor microenvironment to promote the aggregation and functional activation of immunosuppressive populations, including myeloid-derived suppressor cells. Myeloid-Derived Suppressor Cells (mDSCs) and tumor-associated macrophages (TAMs) promote immune evasion in non-small cell lung cancer by metabolic reprogramming, surface molecule alteration, and inhibitory cytokine secretion, all of which upregulate checkpoint ligands such as Programmed Death-Ligand 1 (PD-L1) and CD70, weaken IFN-γ-dependent immune surveillance, and impair T cell cytotoxicity. Among the CRP flavonoid constituents, hesperidin and nobiletin stand out for their ability to modulate the epithelial-mesenchymal transition process through multiple molecular targets and signaling pathways, hence showing very promising potential in the regulation of the lung cancer tumor microenvironment (40).

In TGF-β1 induced EMT models, A549 and H1299 lung adenocarcinoma cells were used as experimental cells, and after determining non - cytotoxic concentrations by MTT assay, the authors systematically applied immunofluorescence, Western blotting, RT - PCR, Transwell migration assays, cell adhesion assays, scratch assays, and SBE-luciferase reporter plasmid transfection to show that nobiletin (10-20 µmol·L^-1^) has a clear effect. Nobiletin can inhibit Smad3 transcriptional activity without interfering with Smad2/3 phosphorylation or nuclear translocation, and suppress Src/FAK/paxillin adhesion signaling by downregulating Zinc finger E-box Binding homeobox 1 (ZEB1) and Matrix Metalloproteinase-2 (MMP-2), MMP-9 expression, thereby blocking lung cancer cell migration, invasion, and adhesion. Sma and Mad-related protein 2 (Smad2) siRNA, Smad3 siRNA, and PcDNA3.1-Flag-Smad3 transfections were used to confirm this (41). The experiments described above conclusively established Smad3 as the important target, showing that nobiletin can restore E-cadherin promoter expression and inhibit N-cadherin promoter increase, while Smad downregulation relieves β-catenin suppression, thus stabilizing the epithelial phenotype. Experiments with A549 cells were performed using MTT assays, circularity quantification, and ELISA detection to examine hesperidin’s effects. Hesperidin (20-40 μmol·L^-^¹) can reduce α-SMA, MMP-9/TIMP-1 ratios, and increased E-cadherin levels, thereby attenuating EMT severity without affecting cell circularity values, which implies that it has limited morphological reversal capacity. In addition, under hypoxic microenvironment conditions, nobiletin was also shown to upregulate miR-200b, suppress downstream Notch-1 and PI3K/Akt axis activation, regulate EMT-related proteins, reverse the EMT-induced immunosuppressive microenvironment, reactivate antitumor immunity, and thus improve treatment sensitivity (42).

From the existing literature, it is clearly established that CRP flavonoid constituents regulate extracellular matrix remodeling and interfere with EMT processes in the tumor microenvironment via several well-defined signaling pathways: TGF-β1/Smad3/β-catenin, Notch-1/miR-200b/PI3K/Akt, C-X-C motif chemokine ligand 12 (CXCL12)/C-X-C chemokine receptor 4 (CXCR4), NF-κB/JNK/MMP9, and STAT1/STAT3/CAF, thus offering multi-target intervention strategies for lung cancer metastasis chemoprevention (43).

However, it must be acknowledged that current studies are predominantly based on cell lines A549 and H1299 and their induced models have not been validated in primary tumor cells or human tumor tissues, and existing studies on *in vivo* experiments mainly employ nude mouse tail vein injection of A549 cells or Lewis cell metastasis models and A549-Luc subcutaneous xenograft models, none of which properly replicate orthotopic lung cancer occurrence or the complex cellular composition of the tumor microenvironment. Meanwhile, there is currently no *in vivo* validation of mDSCs, TAMs, and other immunosuppressive populations, as well as their PD-L1, CD70 checkpoint ligand regulation, mechanistic studies have mostly focused on single pathway blockade without adequately investigating crosstalk and synergistic effects among the TGF-β/Smad3/β-catenin, Notch-1/miR-200b/PI3K/Akt, CXCL12/CXCR4, NF-κB/JNK/MMP9, and STAT1/STAT3/CAF pathways. Additionally, the lack of effect on cell roundness values of hesperidin suggests it’s limited role in morphological reversal, and the molecular mechanism by which nobiletin inhibits Smad3 remains to be further elucidated (44–46).

### 3.5 Angiogenesis suppression to remodel TME

CRP flavonoid constituents modulate the antitumor immune microenvironment by regulating vascular growth, thus showing multi-target intervention potential in lung cancer therapy. The antiangiogenic and antitumor activities of Sinensetin have been confirmed in lung adenocarcinoma models, with supporting evidence from colony formation, Transwell migration-invasion, and tube formation assays. The constituent inhibited A549 cell proliferation, migration, invasion, vascular generation capacity, and suppressed tumor stem cell properties, and the underlying mechanism was elucidated. Sinensetin can downregulate VEGFA transcription by inducing miR-374c-5p expression. Luciferase reporter gene experiments then directly confirmed that miR-374c-5p binds to VEGFA, thereby inhibiting the VEGFA/VEGFR2/AKT.

Meanwhile, sinensetin could directly suppress VEGFR2 phosphorylation levels, blocking downstream signal transmission. *In vivo*, BALB/c nude mice receiving A549 cell inoculation were administered sinensetin at 40 mg·kg^-1^·d^-1^ by gavage for 21 days; results showed suppressed tumor growth and vascular generation, with reduced VEGFA, VEGFR2, and phosphorylated AKT (ser473) protein levels. Sinensetin suppresses recruitment and immunosuppressive function of myeloid-derived suppressor cells and regulatory T cells by lowering VEGF levels, thereby weakening VEGF-VEGFR signal mediated tolerance phenotype induction in T cells, regulatory T cells, and dendritic cells, and thus disrupts vascular-immune barriers in the tumor microenvironment, promoting cytotoxic T lymphocyte infiltration and effectively reversing immune evasion (47).

Nobiletin suppresses vascular generation through multiple mechanisms. First, it inhibits the Akt/HIF-1α pathway, thereby decreasing VEGF expression, as shown in ovarian cancer models for vascular generation and tumor growth control. Second, it blocks STAT3 DNA binding activity, which disrupts STAT3-mediated signaling pathways, reduces angiogenesis-related marker expression, and prevents tumor vascular network formation. Third, it downregulates SREBP1 expression, resulting in decreased lipid synthesis in the tumor microenvironment, improved intratumoral oxygenation, relief of hypoxia, enhanced infiltration of effector T cells and antitumor immune cells into the tumor core, and ultimately stronger antitumor immunity (48).

Several separate studies have demonstrated that hesperidin in combination with the chemotherapeutic agent bleomycin has synergistic antitumor effects in A549 cells. Colony formation and cell migration assays showed clear inhibition of colony formation and cell migration, VEGF levels were suppressed, thus confirming antiangiogenic activity, apoptosis-related markers p53, p21, Bax, and cleaved PARP were all significantly upregulated, caspase-3 activity was directly measured and found to be increased, and autophagy marker Beclin-1 was also upregulated. Therefore, the combined treatment enhances antiproliferative, proapoptotic, antiangiogenic, and autophagy-inducing effects compared with bleomycin alone (49).

From the present studies, it is shown that CRP flavonoid constituents have lung cancer therapeutic effects via several signaling pathways, involving miR-374c-5p/VEGF-A/VEGFR-2/AKT, Akt/HIF-1α/VEGF, STAT3/angiogenesis markers, SREBP1/lipid metabolism/hypoxia, and p53/p21/Bax/caspase-3, which together mediate vascular generation suppression, immune barrier disruption, immunosuppression relief, and tumor microenvironment metabolic remodeling.

Although the existing literature discusses chemotherapy sensitization, it is important to recognize serious limitations of the cell models used: studies on sinensetin, nobiletin, and hesperidin have mainly employed single A549 lung adenocarcinoma cell lines, without validation in other lung cancer subtypes (squamous cell carcinomar) or primary tumor cells, hence extrapolation to general lung cancer populations must be done with caution. Meanwhile, sinensetin *in vivo* experiments were done in BALB/c nude mice, therefore it is not possible to directly evaluate the effects of sinensetin on VEGF-mediated T cell, regulatory T cell, and dendritic cell function in intact immune systems. Likewise, nobiletin’s mechanisms for improving intratumoral oxygenation and immune cell infiltration were validated only in ovarian cancer models, not in lung cancer orthotopic transplant or spontaneous tumor models, so tissue specificity and model complexity remain major concerns.

### 3.6 Potential applications of ferroptosis in lung cancer

Ferroptosis, an iron-dependent form of programmed cell death initiated and executed by lipid peroxidation, has become a hot topic in tumor therapy research in recent years. Its occurrence depends on intracellular ferrous iron overload, membrane lipid oxidative damage, and collapse of antioxidant defenses, and the two most important regulatory nodes are the functional inactivation of glutathione peroxidase 4 and the cystine/glutamate antiporter System Xc-. Evidence supported that nobiletin utilizes this pathway to enhance chemosensitivity in drug-resistant colon cancer cell lines, as it was shown that combination with 5-fluorouracil or doxorubicin greatly suppressed viability and colony-forming capacity in HCT15/5-FU and HCT116/ADR resistant strains, accompanied by downregulation of P-glycoprotein and therefore reduced drug efflux. The study showed evidence for glutathione depletion, malondialdehyde and reactive oxygen species accumulation, mitochondrial cristae loss, and increased membrane density, all of which were reversibly inhibited by Liproxstatin -1. Consistent with this, Western blot analysis revealed that glutathione peroxidase 4, solute carrier family 7 member 11, and ferroportin were downregulated, while divalent metal transporter 1 was upregulated. Bioinformatic screening revealed 16 overlapping genes among colon cancer, ferroptosis, and nobiletin datasets, and functional enrichment analysis showed that positive regulation of Wnt signaling and the RAS pathway is involved in resistance development, whereas survival analysis clearly demonstrated that high NADPH Oxidase 4 (NOX4) and Tissue Inhibitor of Metalloproteinase-1 (TIMP1) expression predicts poor prognosis, and both are downregulated by nobiletin. Therefore, they are strong candidates for reversing drug resistance (50).

Naringenin has been shown to induce ferroptosis-mediated antitumor activity in osteosarcoma via the STAT3/Microsomal Glutathione S-Transferase 2 (MGST2) axis, and thus it is appropriate to elaborate that signal transducer and activator of transcription 3 is a major transcriptional regulator whose sustained activation is intimately linked to tumor progression and immune suppression, whereas microsomal glutathione S-transferase 2, although known for its role in chemotherapeutic drug response, had no known function in osteosarcoma ferroptosis. The authors used CCK8, colony formation, and Transwell assays to show that naringenin inhibited osteosarcoma cell proliferation, colony formation, migration, and invasion, and then they employed reactive oxygen species probes, ferrous iron colorimetry, thiobarbituric acid reactive substance detection, and transmission electron microscopy to demonstrate that the treated cells had an oxidative stress burst. The study first demonstrated that labile iron pool expansion, lipid peroxidation damage, and mitochondrial ultrastructural disruption were all inhibited by ferroptosis inhibitors, then used bioinformatic analysis combined with quantitative real-time PCR and Western blot to show that MGST2 is overexpressed in tumor tissue and is correlated with ferroptosis sensitivity. Subsequent small interfering RNA-mediated gene silencing and Covelin rescue experiments established that naringenin induces tumor cell ferroptosis by suppressing STAT3 transcriptional activity to downregulate MGST2 expression. Finally, nude mouse xenograft models with histochemical staining confirmed the compound’s pro-death effects on solid tumors without overt toxicity to normal organs (51).

Since all the available evidence comes from colon cancer and osteosarcoma models, it is clear that the antitumor activity, ferroptosis-inducing capacity, and molecular targets of nobiletin in lung cancer cell lines, animal models, and clinical samples have not yet been experimentally established.

### 3.7 Potential applications of the cGAS-STING pathway in lung cancer

The cyclic GMP-AMP synthase (cGAS)-stimulator of interferon genes (STING) pathway is a central regulator of cellular innate immune responses and therefore constitutes a major node in tumor immune microenvironment remodeling, since it recognizes cytoplasmic abnormal double-stranded DNA and thereby initiates downstream signaling events that directly and effectively inhibit tumor cell proliferation (52).

Naringin induces apoptosis and blocks immune evasion by regulating a particular pathway, and experimental evidence for naringin-mediated regulation of this pathway has already been established in tumor cell models, with human breast cancer MDA-MB-231 cells serving as the primary experimental system. Experimental groups included control, naringin low/medium/high dose groups (50, 100, 200 μmol·L^-1^), cGAS-specific inhibitor RU.521 group (1 μmol·L^-1^), and combined high-dose naringin plus RU.521 treatment group; cells in each group received corresponding drug treatment for 48 hours before indicator detection. From the results it was shown that naringin could upregulate intracellular cGAS and STING protein expression levels in a concentration-dependent manner; concurrently significantly reducing EdU-positive rates, OD450 values, and proliferating cell nuclear antigen (PCNA) protein expression to achieve tumor cell proliferation suppression, while elevating cell apoptosis rates and p53 protein expression to induce tumor cell apoptosis; furthermore, naringin could downregulate B7 homolog 1 (B7H1), programmed cell death protein 1 (PD-1), and other immune checkpoint molecule expression, effectively blocking tumor cell immune evasion processes. Following 48-hour co-incubation of MDA-MB-231 cells treated with different naringin concentrations with NK cells, granzyme B, tumor necrosis factor-α (TNF-α), and interferon-γ (IFN-γ) expression levels in co-incubation system supernatants and NK cell cytotoxicity against tumor cells were both markedly enhanced, whereas cGAS inhibitor RU.521 could completely reverse naringin-mediated biological effects described above, confirming the cGAS-STING pathway as the critical target for naringin-mediated antitumor proliferation, proapoptotic, and immunomodulatory effects (53).

It is important to recognize that all present experimental studies on naringin activation of the cGAS-STING pathway have been carried out using breast cancer MDA-MB-231 cell models, and therefore there is no direct experimental evidence from lung cancer-related cell lines, lung cancer animal models, or clinical lung cancer samples. Consequently, whether the cGAS-STING pathway is a valid target for CRP flavonoid regulation of lung cancer immune microenvironments, and the exact regulatory mechanisms and dose-effect relationships of nobiletin, hesperidin, tangeretin, and other CRP flavonoid monomer constituents on this pathway in lung cancer cells, remain to be established.

### 3.8 Interplay among signaling pathways

#### 3.8.1 PI3K/AKT/mTOR as a core pathway regulating cell proliferation and apoptosis

**Figure 1:**
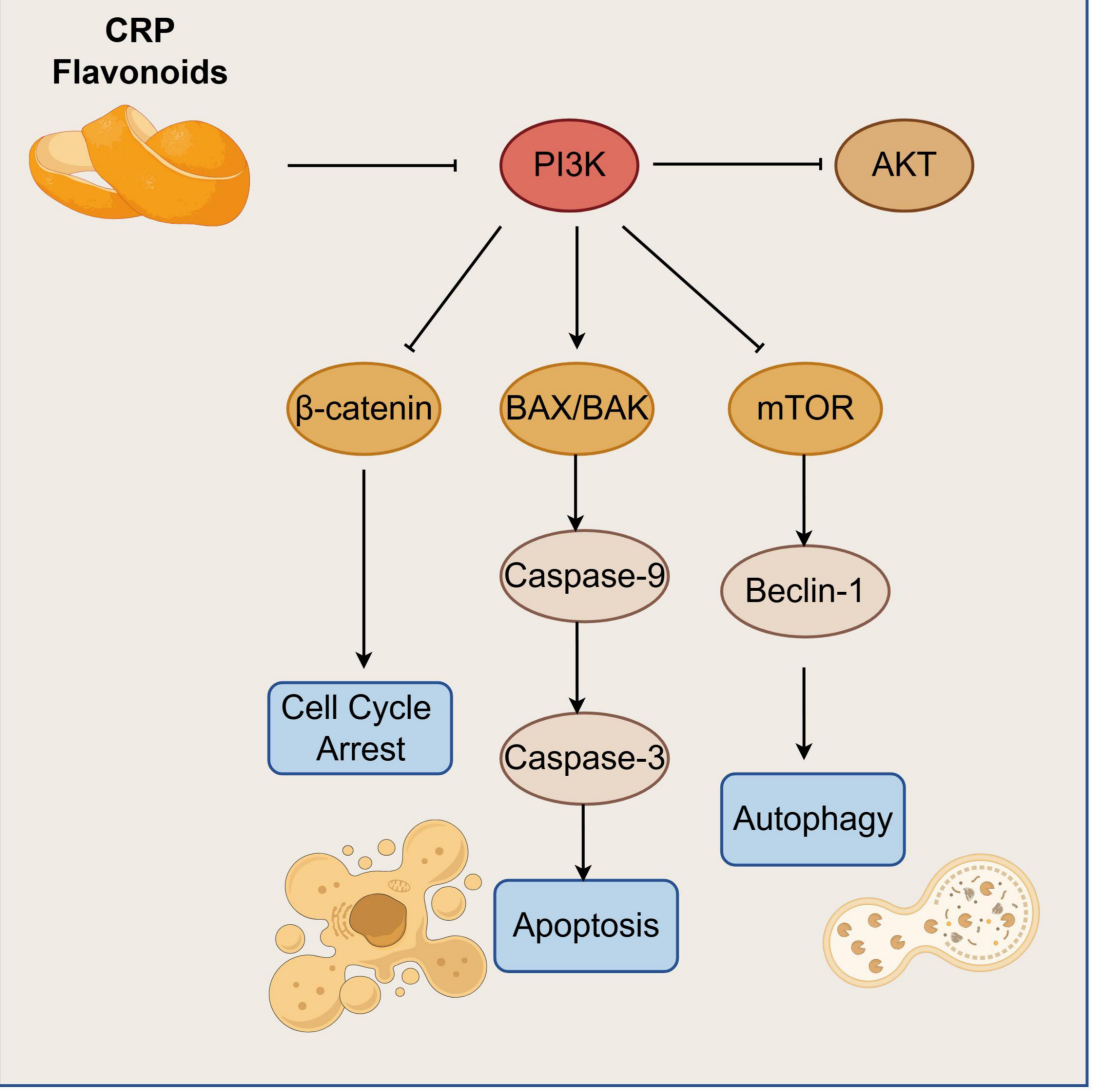
PI3K/AKT/mTOR as a core pathway regulating cell proliferation and apoptosis.

In lung adenocarcinoma models, CRP flavonoids inhibit β-catenin nuclear translocation and downstream proliferation-related gene transcription by suppressing the AKT/β-catenin pathway, thereby inducing cell cycle arrest, and at the same time, they downregulate Akt/mTOR signal transduction, thus affecting cell metabolism and survival. Importantly, when PI3K/AKT pathway activity is suppressed by CRP flavonoids, phosphorylation-mediated inhibition of proapoptotic factors occurs. Optic protein Bcl-2-associated Death promoter (BAD) is relieved in such a way that it promotes Bcl-2-associated X protein (BAX)/Bcl-2 antagonist/killer (BAK)-mediated mitochondrial outer membrane permeabilization, hence facilitating release of cytochrome c to activate the caspase -9/-3 cascade and drive cell apoptosis, but at the same time it suppresses mTOR-mediated regulation of ULK complexes, thereby modulating autophagy pathway activation. Therefore, the precise outcome of this dual regulation depends on the specific CRP flavonoid constituent, its applied concentration, and the cellular microenvironmental context. Importantly, low-concentration hesperidin enhances apoptosis sensitivity via moderate autophagy activation, whereas high-concentration nobiletin induces pyroptosis via excessive autophagy. As shown in **Figure 1**.

#### 3.8.2 Tumor immune microenvironment regulation networks through multiple routes

**Figure 2:**
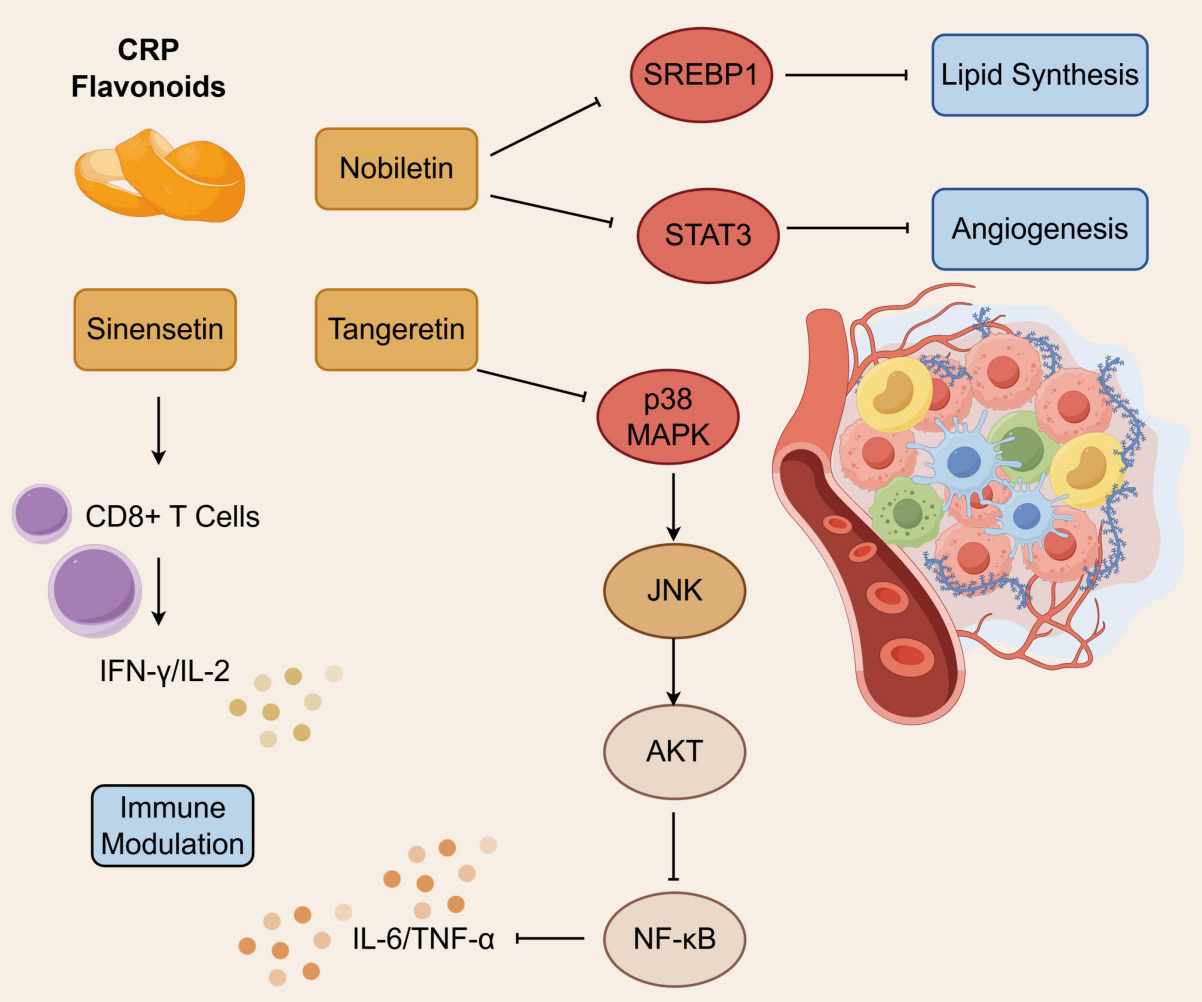
Tumor immune microenvironment regulation networks through multiple routes.

The cGAS-STING pathway plays an important role in tumor immune regulatory systems, which can stimulate NF-κB activation and transcription processes through upstream STING regulatory effects, thereby mediating pro-inflammatory response generation. As a key mediator of organismal immune responses, when deficient of STING would promotes tumor cell immune evasion and thus malignant tumor proliferation (54, 55). Since it mediates pro-inflammatory response generation, this pathway can be grouped together with oxidative stress regulation, MAPK-NF-κB-STAT3 pathway-mediated inflammatory suppression, PI3K/AKT/mTOR pathway blockade, and immune evasion regulation, all of which were the fundamental components of tumor microenvironment remodeling that contribute to tumor cell damage response. p38 MAPK and JNK are members of the MAPK family, which represent important targets for CRP flavonoid intervention in tumor inflammatory microenvironments. It is clear from experiments on lung cancer A549 cells that IL-1β activates p38 MAPK, JNK, and AKT, leading to COX-2 expression, while tangeretin inhibits NF-κB activation by blocking their phosphorylation, thus reducing IL-6, TNF-α, and other pro-inflammatory mediators. Nobiletin suppresses STAT3 DNA binding activity, thus inhibiting angiogenesis-related marker expression, and at the same time suppresses SREBP1 nuclear translocation, which in turn reduces fatty acid synthesis, lowers lipid accumulation, and ameliorates tumor microenvironmental hypoxia. Importantly, sinensetin enhances CD8^+^T cell cytotoxicity and increases IFN-γ and IL-2 secretion in lung cancer models. Thus, tumor cell proliferation-apoptosis regulation and tumor immune microenvironment improvement are the core targets on anti-lung cancer of CRP flavonoid. As presented in **Figure 2**.

#### 3.8.3 Coordinated suppression networks of EMT and angiogenesis

**Figure 3:**
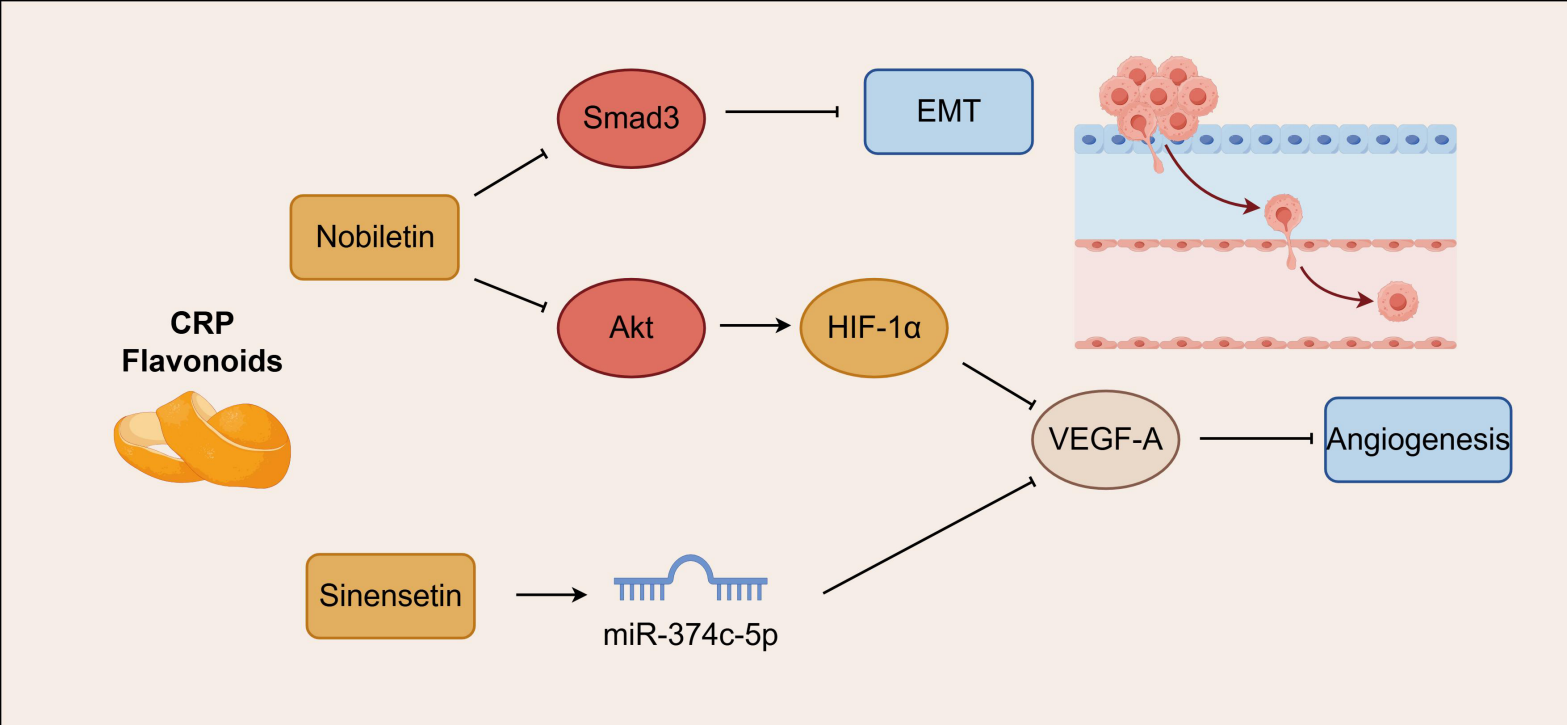
Regulatory networks of oxidative stress-inflammation-metabolic reprogramming.

The TGF-β1/Smad3 pathway serves as a central node for CRP flavonoid-mediated EMT regulation, whereas angiogenesis is predominantly governed by the HIF-1α/VEGF axis. Matrix metalloproteinases (MMPs), functioning as enzymatically active proteolytic proteins, constitute critical factors in basement membrane degradation. Specifically, MMP-9 generates active TGF-β precursors through proteolytic processing and concurrently modulates VEGF to facilitate tumor angiogenesis and subsequent metastatic colonization. Epithelial-mesenchymal transition (EMT), orchestrated by transcriptional regulators including slug, Twist, ZEB1, and ZEB2 during cellular migration and invasion, is further promoted by TGF-β1-activated Smad3, which induces the secretion of MMP-2 and MMP-9 to participate in extracellular matrix remodeling. VEGF, acting as a pivotal angiogenic factor, drives endothelial cell proliferation and suppresses T-cell infiltration when overexpressed. Nobiletin specifically targets Smad3 transcriptional activity, downregulating EMT-associated transcription factors and MMP expression, while simultaneously attenuating VEGF production through Akt/HIF-1α pathway inhibition, thereby achieving dual intervention against EMT and angiogenesis. In contrast, sinensetin suppresses angiogenesis via miR-374c-5p-mediated VEGF-A silencing coupled with VEGFR-2/AKT signaling blockade. Whether synergistic interactions exist between these two constituents, and what underlying molecular cross-talk mechanisms might operate, remain to be established through direct experimental validation (56). As illustrated in **Figure 3**.

#### 3.8.4 Regulatory networks of oxidative stress-inflammation-metabolic reprogramming

**Figure 4:**
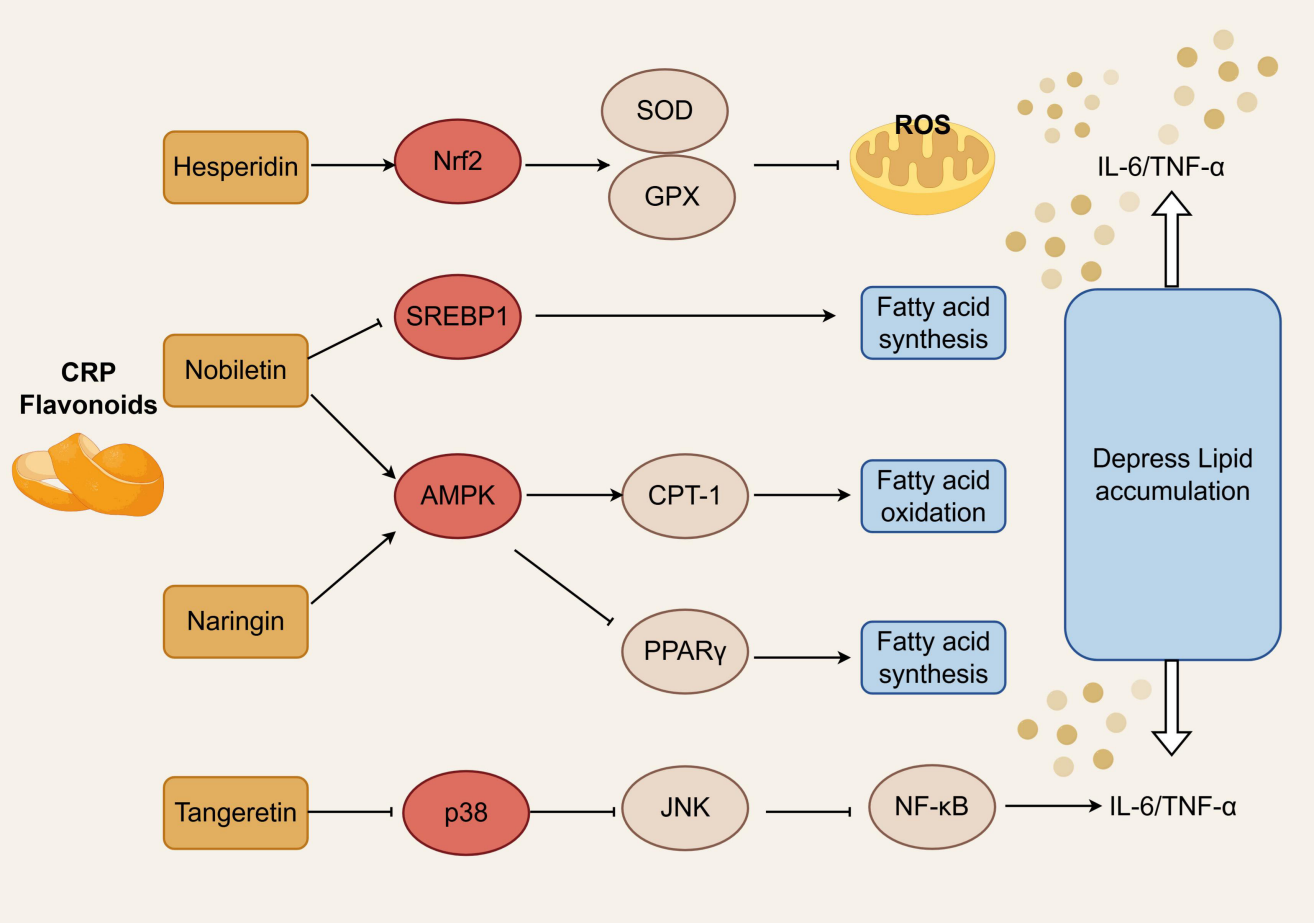
Regulatory networks of oxidative stress-inflammation-metabolic reprogramming.

Antioxidation and suppression of lipid metabolic reprogramming constitute key mechanisms underlying the remodeling of this microenvironment by CRP flavonoids. Nobiletin blocks fatty acid biosynthesis through SREBP1 nuclear translocation suppression, and promotes fatty acid catabolism through AMPK-mediated carnitine palmitoyltransferase 1 upregulation, improving intratumoral oxygenation status. Naringin activates Nrf2 pathway, upregulating superoxide dismutase and glutathione peroxidase activities, lowering lipid peroxidation levels; concurrently through AMPK pathway-enhanced fatty acid β-oxidation, suppressing PPARγ transcriptional activity, reducing lipid synthesis and accumulation, lowering lipotoxicity-mediated ROS generation, and reducing ROS-mediated NF-κB activation. In lung cancer models, tangeretin can suppress NF-κB activation and IL-6, TNF-α, and other inflammatory factor release effects, thereby improving antitumor immune microenvironments. Whether these anti-inflammatory mechanisms and metabolic regulatory effects represent different targets of the same constituent, or complementary actions of different constituents, remain unvalidated through experiments. As depicted in **Figure 4**.

## 4 The potential for clinical translation

### 4.1 Boosting efficacy of immune-checkpoint inhibitors

The flavonoids in Citri Reticulatae Pericarpium modulate immune signaling and immune-cell behavior by acting on multiple pathways. Naringin activates CD169^+^ macrophages in draining lymph nodes, raising the expression of CD169, IL-12 and CXCL10, which recruits cytotoxic T lymphocytes to the tumor bed and promotes their activation, thereby intensifying the antitumor response. Nobiletin lowers PD-L1 levels in non-small-cell lung cancer cells by interfering with the EGFR/JAK2/STAT3 axis. Within the JAK/STAT cascade, extracellular stimuli engage cell-surface receptors, prompting JAK activation and subsequent STAT phosphorylation; phosphorylated STAT proteins then enter the nucleus and boost PD-L1 transcription, increasing membrane PD-L1 abundance. Agents that block or dampen this cascade interrupt the signaling relay, prevent PD-L1 up-regulation, and can thus improve the efficacy of immune-checkpoint inhibitors (57, 58). As presented in **Table 2**.

**Table 2:** CRP flavonoid nano-carrier systems.

Carrier platform	Core design	Key findings	Translational barriers	References
tFNAs	Tetrahedral DNA loading	Improved cellular uptake and sustained-release effects; enhanced bioavailability	Immunogenicity, *in vivo* degradation kinetics, and scale-up production processes undefined	(59)
ZIF-zni	pH-responsive metal-organic framework loading	Enhanced cytotoxicity; good carrier biocompatibility	Degradation product toxicity, batch stability, and tumor pH heterogeneity effects unassessed	(60)
Hsp-AuNPs	Gold nanoparticle electrostatic assembly	Improved antitumor activity	Long-term tissue accumulation, oxidative stress effects, and clinical translation costs unevaluated	(61)
CaFe_2_O_4_-PR	Magnetic casein-ferric calcium dual-targeting carrier	Enrichment at target sites; hypothesized synergistic antitumor effects	Magnetic field parameter standardization, receptor expression heterogeneity, and oxidative stress effects unexamined	(62)
NLC	Solid lipid nano-structure co-loading alectinib-hesperidin, inhalation administration	72h release improved, AUC increased	Validated only in A549; rescue experiments not conducted	(63)
Nanoemulsion	Oil-water nanoemulsion co-loading naringin-celecoxib, atomization inhalation	Alveolar deposition; detectable distribution in brain/liver/bone metastatic foci	Pathological state effects on deposition unmodeled; tumor targeting efficiency unvalidated	(64)

### 4.2 Development of novel drug delivery systems

Since the major bottleneck in natural product drug development is poor solubility, pronounced first-pass effects, and inadequate targeting capacity, researchers have designed several nano-carrier systems for CRP flavonoids and systematically optimized their *in vivo* fate by tuning particle size, surface charge, and responsive release properties.

#### 4.2.1 Nucleic acid-based carriers

Since tetrahedral framework nucleic acids (tFNAs) are tetrahedral DNA nanostructures possessing natural cell membrane penetration ability, loading nobiletin onto them allows one to overcome the poor water solubility and poor plasma stability of nobiletin, thus greatly improving cellular uptake efficiency and permitting sustained release (59).

#### 4.2.2 Metal-organic frameworks

Because zeolitic imidazolate frameworks--zni (ZIF-zni) has pH sensitivity, it can achieve selective drug release in tumor acidic microenvironments, and therefore hesperetin-loaded ZIF-zni showed enhanced cytotoxicity at a concentration of 40 μg·mL^-1^, without loss of biocompatibility (60).

#### 4.2.3 Inorganic nanoparticles

Since hesperidin-Ultrasound nanoparticles (Hsp-AuNPs) employ electrostatic assembly of hesperidin with glutathione and have gold core plasmonic properties, they exhibit pH-dependent release behavior in breast cancer models and show antitumor activity that is better than that of the free drug (61).

#### 4.2.4 Magnetic composite carriers

Because Casein-calcium ferriter (CaFe2O4) nanohybrid carrier possess both magnetic properties and receptor-targeting ability, progesterone receptor-mediated endocytosis together with external magnetic field guidance can efficiently deliver hesperidin to the target site, thereby producing excellent carrier - drug synergistic antitumor effects (62).

#### 4.2.5 Lipid-based carriers

Alectinib and Hesperidin (ALB-HSD NLC) is a solid lipid-based nanocarrier prepared by melt emulsification-ultrasonication, which released 2.5 fold more drug at 72 hours than the corresponding suspension, and in A549 cells, the IC_50_ values of alectinib and hesperidin were 2.289 µg·mL^-1^ and 73.52 µg·mL^-1^ respectively, with a greatly reduced toxicity index for NLC. Pharmacokinetic studies showed Area Under the Curve from time 0 to time t (AUC0-t) increases of 1.38 and 1.57 fold for both components, and importantly, this formulation demonstrated positive antitumor efficacy in syngeneic models (63).

#### 4.2.6 Emulsion-based carriers

Naringin/celecoxib co-loaded nanoemulsion has a particle size of 75–106 nm and gives a median mass aerodynamic diameter <5 μm after atomization, hence it deposits efficiently in the alveoli. More importantly, it distributes in the brain, liver, bone, and other metastatic target organs, which makes it a very promising candidate for treating both primary lesions and metastatic foci, although its targeting efficiency under tumor conditions still needs experimental validation.

Although carrier design rationality is well supported by preclinical data, it is indisputable that several important translational medicine issues remain unresolved: namely, tFNA immunogenicity, ZIF *in vivo* degradation product toxicity, gold nanoparticle long-term tissue accumulation, and magnetic particle oxidative stress effects all lack rigorous toxicological evaluation (64).

## 5 Summary and prospects

### 5.1 Summary of research findings on CRP flavonoid regulation of lung cancer TME

This review systematically examined molecular mechanisms whereby CRP flavonoid constituents regulate lung cancer tumor microenvironments. Available evidence indicates that rather than directly killing tumor cells through single targets, these compounds reshape TME through multi-component, multi-pathway coordinated patterns, exerting antitumor effects indirectly. Core constituents including nobiletin, hesperidin, tangeretin, naringin, and sinensetin operate through six primary routes: (1) Oxidative stress and inflammatory response modulation. Most constituents activate Nrf2/ARE pathways and suppress NF-κB pathways to reduce ROS and pro-inflammatory mediator levels, whereas naringenin demonstrates pro-oxidative stress effects inducing apoptosis in A549 cells. (2) Correction of lipid metabolic reprogramming. Through AMPK/SREBP-1c pathways suppressing fatty acid synthesis and promoting β-oxidation, reversing immune suppression triggered by metabolic disturbance. (3) Pyroptosis induction. Nobiletin promotes Caspase-1/GSDMD-mediated pyroptosis through miR-200b/JAZF1/NF-κB/NLRP3 pathways, enhancing CD4^+^ and CD8^+^ T cell infiltration. (4) EMT suppression. Nobiletin and hesperidin target TGF-β1/Smad3, Notch-1/miR-200b/PI3K/Akt and other pathways to block tumor invasion and metastasis. (5) Angiogenesis suppression. Sinensetin and nobiletin coordinately suppress vascular generation through miR-374c-5p/VEGF-A/VEGFR-2/AKT and Akt/HIF-1α/VEGF pathways respectively. (6) ICI efficacy enhancement. Nobiletin downregulates PD-L1, while naringin activates CD169^+^ macrophages to recruit cytotoxic T lymphocytes. Furthermore, novel nano-delivery systems including tFNAs and ZIF-zni have improved CRP flavonoid defects of poor water solubility and low bioavailability, providing technical support for clinical translation. Nobiletin and naringenin ferroptosis-inducing effects, and naringin cGAS-STING pathway activation, have been confirmed in other cancer types but remain unvalidated in lung cancer models. The overall mechanism of flavonoids in tangerine peel is shown in **Table 3**.

**Table 3:** Mechanism of action of CRP flavonoids.

Compound	Biological processes	Pathway	Key findings	Limitations	References
Nobiletin	EMT inhibition	TGF-β1/Smad3/ZEB1	10-20 µmol·L^-1^: E-cadherin promoter restored, N-cadherin promoter inhibited	A549/H1299 only;	(41)
		MMP-2/MMP-9/E-cadherin	Smad3 siRNA mimics effect, overexpression attenuates	Smad3 mechanism unclear;	(41)
	Anti-angiogenesis	Akt/HIF-1α/VEGF	Nude mice: 20/40 mg·kg^-1^, lung nodules decreased	Ovarian cancer data extrapolated to lung cancer	(48)
	Lipid metabolism reprogramming	SREBP-1/AMPK/CPT1	Intratumoral oxygenation improved; fatty acid synthesis decreased, fatty acid oxidation increased	—	(28, 29)
	Pyroptosis	miR200b/JAZF1/NF-κB/NLRP3/caspase-1/GSDMD	CD4^+^/CD8^+^ T cell infiltration increased; IL-1β decreased	NLRP3 dual role not clarified	(34)
		MDSCs/TAMs/ PD-L1/CD70	suppress immune escape in non-small cell lung cancer	BALB/c nude mice immunodeficient; MDSCs/TAMs/PD-L1/CD70 not validated *in vivo*	(35)
Naringin	Anti-oxidative stress	Nrf2/SOD/GPx	DEN/2AAF rats: Nrf2 increased, GSH increased, lipid peroxidation decreased, TNF-α/IL-1β/IL-6 decreased, IL-10 increased	Rat chemical-induced model differs from human lung cancer; nanocomposite used	(16)
	Lipid metabolism reprogramming	AMPK/PPARγ/CPT1	Fatty acid β-oxidation promoted; cholesterol homeostasis maintained	Nanocomposite *in vivo* distribution, clearance and toxicity not evaluated	(25–27)
	Anti-inflammatory	NF-κB/iNOS	NF-κB/iNOS decreased; inflammatory cytokine balance regulated	Optimal dose and bioavailability data insufficient	(17)
	Pro-oxidative stress	ROS/SOD/MDA/Nrf2/NQO1/HO-1	A549: 30-120 µmol·L^-1^, ROS increased, SOD decreased, MDA increased, proliferation inhibited, apoptosis increased	Contradicts antioxidant effects of most citrus flavonoids; *in vitro* only; no normal cell control; concentration gradient insufficient	(17)
Hesperetin	Lipid metabolism reprogramming	CYP1A1/CYP1B1	DNA damage active intermediates decreased; IL-8 decreased	Specific experimental model unclear; lacks detailed *in vivo* validation; no head-to-head comparison with other flavonoids	(30)
	EMT inhibition	CXCL12/ CXCR4	Migration and invasion ability decreased; NF-κB/JNK blocked	No primary tumor cell validation; lacks MDSCs/TAMs verification	(44)
	Anti-inflammatory	NF-κB/JNK/MMP-9	A549: 20-40 µmol·L^-1^, α-SMA decreased, MMP-9/TIMP-1 decreased, E-cadherin increased	Morphological reversal limited (circularity value unchanged); cross-talk between pathways not explored	(13)
	Anti-angiogenesis	VEGF	Combined with bleomycin: VEGF decreased, p53/p21/Bax/cleaved PARP increased, Beclin-1 increased, caspase-3 activated, synergistic effect	Dose-response relationship unclear *in vivo*; autophagy role in lung cancer not clarified	(49)
Sinensetin	Anti-inflammatory	AKT/β-catenin pathway	downregulated N-cadherin, VEGFA, and AKT/β-catenin pathway expression, while upregulating E-cadherin and IFN-γ levels	A549 and H1299 cells only	(16)
	Anti-angiogenesis	miR-374c-5p/VEGF-A/VEGFR-2/AKT	Nude mice: 40 mg·kg^-1^×21d, tumor growth and angiogenesis decreased, VEGF-A/VEGFR-2/p-AKT decreased	Only A549 cell line; nude mice immunodeficient; lacks other lung cancer subtypes validation	(47)
Tangeretin	Anti-inflammatory	p38 MAPK/JNK/AKT/NF-κB/COX-2	Polyurethane-induced mice: MPO decreased, ICAM-1/IL-6/TNF-α/NF-κB decreased; IL-1β-induced A549: COX-2 decreased	Non-lung cancer model (polyurethane is inflammation/fibrosis model); ERK activation not suppressed; lacks lung cancer orthotopic model	(14)
		JAK/STAT3/MMPs/caspase-3	JAK/STAT-3 phosphorylation decreased, MMPs decreased, caspase-3 increased, COX-2 transcription inhibited	*In vitro*-*in vivo* consistency needs verification; lacks normal cell control; concentration-effect relationship unclear	(14)

### 5.2 Contradictory findings and context-dependent effects

Current research contains multiple contradictory findings, indicating that antitumor effects of CRP flavonoids are highly context-dependent and require careful interpretation. First, most constituents demonstrate antioxidant properties, whereas naringenin at 30-120 µmol·L^-1^ exhibits pro-oxidative effects in A549 cells, with effect transition points remaining undefined due to lack of standardized concentration gradients and normal cell controls. Second, pyroptosis induction in tumor cells activates immunity, while pyroptosis in dendritic cells impairs antigen presentation, and net effects of nobiletin-mediated suppression in lung cancer models remain unresolved. Third, hesperidin combined with bleomycin can enhance autophagy, yet autophagy in lung cancer possesses both pro-survival and pro-death functions, with relevant dose-effect relationships lacking systematic validation. Fourth, both nobiletin and hesperidin target TGF-β1/Smad3 pathways, but the former suppresses Smad3 transcriptional activity while the latter shows limited capacity for morphological reversal, with molecular target differences and synergistic/antagonistic potential between them undefined. These contradictions demonstrate that CRP flavonoid TME regulatory effects are influenced by multiple factors including compound structure, applied concentration, cellular background, and immune status, creating substantial risk for direct extrapolation of single-model results to clinical settings.

### 5.3 Limitations of current research evidence

Existing studies contain systematic methodological limitations that severely restrict reliability and generalizability of findings. First, *in vitro* studies rely excessively on A549 and H1299 lung adenocarcinoma cell lines, lacking validation in squamous cell carcinoma, small cell lung cancer, and primary cells; *in vivo* studies predominantly use subcutaneous xenograft and immunodeficient mouse models, unable to simulate orthotopic lung cancer pathological characteristics and intact immune microenvironments. Second, most focus on single constituents, lacking head-to-head comparisons; drug administration doses and evaluation indicators are inconsistent, with dose-effect relationships unclear; rescue experiment application is limited, with insufficient target-specific validation. Third, mostly single linear pathway descriptions, lacking pathway cross-talk; context-dependent mechanism studies under hypoxic and acidic microenvironmental conditions are missing. Fourth, currently no studies have employed single-cell sequencing or spatial omics to analyze heterogeneous responses of cell subpopulations; only one study (12) involves untargeted metabolomic analysis, with no multi-omics integrated research, and gut microbiota-mediated flavonoid metabolic activation studies have not yet been reported. Fifth, extract origin and year differences cause large fluctuations in core constituent content, making constituent-effect relationships difficult to establish.

### 5.4 Key barriers to clinical translation

The text focused on the pre-clinical study. Based on the mechanisms of CRP flavonoids in lung cancer treatment, this review explores their potential synergistic effects with ICIs and presents novel drug delivery systems to enhance flavonoid bioavailability. But barriers remain in translating preclinical findings to clinical treatment of lung cancer. First, the core flavonoids of CRP have poor water solubility, with oral bioavailability below 10%. Second, *in vitro* IC_50_ values are mostly at µmol·L^-1^ levels with low potency; conversion from animal doses to human equivalent doses lacks scientific basis; and interactions with chemotherapy and ICIs remain unassessed. Third, Long-term toxicity and accumulation effects studies are absent; nanocarrier immunogenicity and degradation product toxicity lack systematic evaluation. Fourth, currently no clinical trials for lung cancer are registered; human pharmacokinetic characteristics, effective plasma concentrations, and target occupancy remain completely unknown; efficacy biomarkers and individualized dosing strategies remain to be established.

### 5.5 Limitations of this review

This review is a narrative review with the following inherent limitations that readers should interpret carefully. First, strict systematic review search protocols were not employed, potentially resulting in selective inclusion bias; only Chinese and English publicly published literature was covered, and search cutoff was March 2026. Second, included studies are predominantly basic experiments, with no high-quality clinical studies; quality assessment and evidence grading were not conducted, and conclusions represent descriptive synthesis rather than quantitative meta-analysis. Third, conclusions regarding ferroptosis, cGAS-STING pathway regulation, and others are extrapolated from other cancer types, carrying tissue-specific risk; pathway networks have not undergone experimental validation. Fourth, focus on indirect mechanisms of TME regulation, with insufficient discussion of direct antitumor effects; emphasis on modern pharmacology, with limited interpretation of traditional Chinese medicine theory-guided formula compatibility and pattern-based treatment.

### 5.6 Future research directions and prospects

In response to current research limitations and translational barriers, future work should advance around four core themes: (1) Use standardized extracts or monomers, conduct structure-activity relationship studies, develop dose conversion standards and required indicator sets, and improve research comparability. (2) Introduce single-cell sequencing and spatial omics to analyze heterogeneous responses of cell subpopulations; conduct gut microbiota-metabolome joint analysis to clarify microbiota-mediated metabolic activation mechanisms; systematically validate roles of emerging pathways including ferroptosis and cGAS-STING in lung cancer. (3) Establish pathological microenvironment models to clarify effect transition nodes; conduct normal versus tumor cell comparative studies to validate selective toxicity, providing basis for clinical safe drug use. (4) Develop humanized mice and lung cancer organoid-immune co-culture models; complete GLP toxicology and human pharmacokinetic studies for core constituents; explore combination regimens and sequential strategies with ICIs and chemotherapy. (5) Design phase Ib/IIa trials for advanced lung cancer with TME biomarkers and clinical benefit rate as endpoints. (6) Use AI-assisted network pharmacology to predict optimal constituent combinations; develop TME-responsive nanocarriers and explore local delivery strategies such as inhalation administration to improve targeting and bioavailability.

Overall, CRP flavonoids, through multi-component and multi-target advantages, demonstrate significant potential in reshaping lung cancer TME and enhancing antitumor immunity, representing a high-quality candidate direction for adjuvant lung cancer drug development. Future progress requires breaking through druggability barriers step by step through standardized research, deepened mechanistic analysis, systematic preclinical validation, and regulated early-phase clinical exploration, driving translation from basic research to clinical application and providing novel strategies for multi-modal comprehensive lung cancer treatment.
